# Quantifying and correcting for speed and stride frequency effects on running mechanics in fatiguing outdoor running

**DOI:** 10.3389/fspor.2023.1085513

**Published:** 2023-04-17

**Authors:** Marit A. Zandbergen, Jaap H. Buurke, Peter H. Veltink, Jasper Reenalda

**Affiliations:** ^1^ Department of Biomedical Signals and Systems, Faculty of Electrical Engineering, Mathematics and Computer Science, University of Twente, Enschede, Netherlands; ^2^ Department of Rehabilitation Technology, Roessingh Research and Development, Enschede, Netherlands

**Keywords:** marathon, kinematics, inertial measurement unit, biomechanics, acceleration, endurance

## Abstract

Measuring impact-related quantities in running is of interest to improve the running technique. Many quantities are typically measured in a controlled laboratory setting, even though most runners run in uncontrolled outdoor environments. While monitoring running mechanics in an uncontrolled environment, a decrease in speed or stride frequency can mask fatigue-related changes in running mechanics. Hence, this study aimed to quantify and correct the subject-specific effects of running speed and stride frequency on changes in impact-related running mechanics during a fatiguing outdoor run. Seven runners ran a competitive marathon while peak tibial acceleration and knee angles were measured with inertial measurement units. Running speed was measured through sports watches. Median values over segments of 25 strides throughout the marathon were computed and used to create subject-specific multiple linear regression models. These models predicted peak tibial acceleration, knee angles at initial contact, and maximum stance phase knee flexion based on running speed and stride frequency. Data were corrected for individual speed and stride frequency effects during the marathon. The speed and stride frequency corrected and uncorrected data were divided into ten stages to investigate the effect of marathon stage on mechanical quantities. This study showed that running speed and stride frequency explained, on average, 20%–30% of the variance in peak tibial acceleration, knee angles at initial contact, and maximum stance phase knee angles while running in an uncontrolled setting. Regression coefficients for speed and stride frequency varied strongly between subjects. Speed and stride frequency corrected peak tibial acceleration, and maximum stance phase knee flexion increased throughout the marathon. At the same time, uncorrected maximum stance phase knee angles showed no significant differences between marathon stages due to a decrease in running speed. Hence, subject-specific effects of changes in speed and stride frequency influence the interpretation of running mechanics and are relevant when monitoring, or comparing the gait pattern between runs in uncontrolled environments.

## Highlights

• Effects of changes in speed and stride frequency on mechanics are subject-specific
• Speed and stride frequency explain 20%–30% of the variance in PTA and knee angles
• Changes in speed and stride frequency masked fatigue-related changes in mechanics
• Correct mechanics for changes in speed and stride frequency in outdoor running

## 1. Introduction

Motion analysis in running provides objective information about running technique. This information can be used to improve running performance (1, 2), monitor effects of fatigue on the gait pattern (3, 4), and possibly reduce injury risk through real-time feedback on mechanical quantities (5–7). Feedback is often provided on peak tibial acceleration (PTA) since this quantity is easy to measure and provides information about a combined effect of impact forces and running technique on the acceleration of the tibia (8). However, PTA is not directly linked to forces in the body since PTA is unable to represent the contribution of muscle contractions (9, 10). Monitoring of knee angles is of interest due to their role in shock attenuation during running (11) and their tendency to change with running-induced fatigue (9, 10, 12).

Traditionally, running mechanics were measured in a gait laboratory. A laboratory setting allows researchers to control or minimize influences on the gait pattern from, for instance, running speed, inclination, running surface, and the weather. Simultaneously, a laboratory setting is restricted to an artificial environment that is not sport-specific. Multiple mechanical quantities concerning peak accelerations and shock attenuation showed important differences between overground and treadmill running (13–16). Hence, running mechanics should be analyzed in a representative environment since findings from laboratory-based treadmill studies cannot easily be generalized to overground running (14, 17, 18).

One essential difference between treadmill and outdoor running is the ability to adapt running speed. Most runners lower their speed after prolonged running due to fatigue (19, 20). The influence of running speed and stride frequency on mechanical variables has extensively been studied in controlled environments and typically on a treadmill. PTA increases with an increase in running speed (8) or a decrease in stride frequency (21). PTA showed a strong significant linear regression with speed in treadmill running (22, 23). Individual variances in these relationships were large, highlighting the need for subject-specific analysis (22, 24). Additionally, maximum stance phase knee flexion increased with an increase in speed or a decrease in stride frequency (25, 26). No speed effect on knee flexion angles at initial contact was found over a small range of running speeds in recreational runners (26). Hence, running speed and stride frequency influence PTA and knee joint angles in running which makes it hard to compare quantities both within and between runs when speed and stride frequencies are not consistent. Two previous studies corrected mechanical quantities during running in an uncontrolled setting for speed by computing individual ratios (i.e., dividing by speed) (19, 20). This correction assumes that the relationship between speed and quantities of interest crosses the origin (i.e., quantity of interest is zero when the speed is zero) and is linear over the full range of running speeds for all subjects. Such a relationship assumes that an increase in speed of 1 km/h during walking and running will result in the same increase in a quantity of interest. However, in the case of PTA, the regression equation between speed and PTA differs between foot strike patterns (20), between subjects (24) and the intercepts of group-based analyses do not appear to cross the origin (20). Thus, individual ratios likely oversimplify the relationship between speed and quantities of interest.

Inertial measurement units (IMUs) can measure running mechanics in a sport-specific setting and open up new possibilities for real-time feedback on running technique in a representative environment (18). Feedback on PTA values is often used to improve running technique by providing warnings for high PTA values, both in commercial devices and in research (27–31). Additionally, algorithm development allows the estimation of knee angles based on a minimal sensor set (32). Feedback on running technique is often based on an arbitrary fixed threshold independent of running speed and stride frequency which can mask fatigue-related changes in running biomechanics. Without correcting for the effects of speed and stride frequency, the origin of changes is unclear, preventing appropriate interpretation and feedback on running biomechanics. Hence, this study aims to quantify and correct for the subject-specific effect of running speed and stride frequency on changes in impact-related running mechanics during a fatiguing outdoor run.

A marathon was used as an uncontrolled setting to ensure that a wide range of external influences (e.g., fatigue, different surfaces, other runners) found in typical uncontrolled outdoor running were incorporated to improve the ecological validity of relationships. We hypothesized that:

1. Running speed and stride frequency decrease toward the end of the marathon
2. The influence of running speed and stride frequency on PTA, knee angles at initial contact, and maximum stance phase knee flexion angles differs between subjects
3. Correcting PTA and knee angles for subject-specific changes in speed and stride frequency results in significant changes during the marathon which are not found in uncorrected values

## 2. Methods

### 2.1. Participants

Nine healthy recreational runners participated in this study. Technical errors resulted in missing data for two subjects. Therefore, data from three females and four males were included (mean (standard deviation); age: 36 (11) years, height: 181 (5) cm, mass: 74 (8) kg, running experience: 7 (4) years). All subjects gave written informed consent before participating in this study. The Ethics Committee Computer and Information Science of the University of Twente approved the study protocol.

### 2.2. Measurement systems

**Figure 1:**
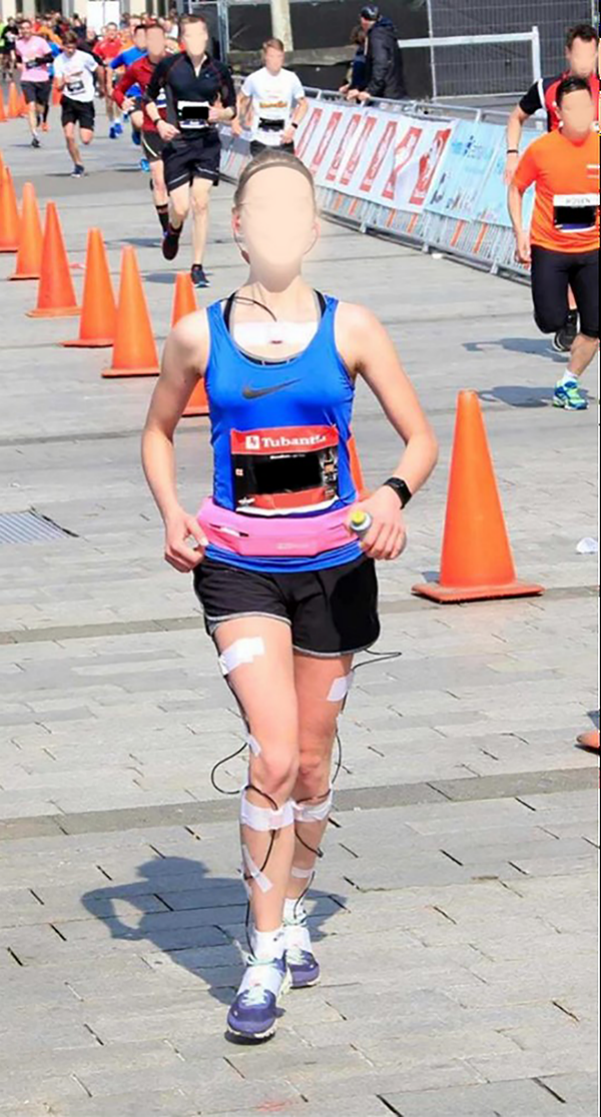
One of the runners a few meters before the finish line. The bodypack and battery pack are placed in the pink belt. White tape is visible, which covers the sensors and fixates sensor wires.

Subjects were equipped with eight IMUs (sampling frequency: 240 Hz, dimensions: 36 × 25 × 10 mm, weight: 16 g, MVN Link, Xsens Technologies, Enschede, The Netherlands). IMUs were placed on the sternum, back of the pelvis, and bilaterally on the midportion of the lateral upper leg, proximally on the tibia, and on the midfoot. Hair on the skin was shaved to improve IMU attachment before IMUs were fixed to the skin with double-sided tape and covered with additional tape. IMUs on the midfoot were placed under the tongue of the shoes. Wires between IMUs were loosely taped to the skin to prevent entanglement, see Figure 1. IMUs were connected with a bodypack and battery pack. The bodypack delivered power from the battery pack to the IMUs and synchronized and stored data from the IMUs on internal memory. The bodypack and battery pack weighed 220 grams (33) and were placed in a neoprene storage belt around the waist of the runners. Subjects used their personal sports watches with a global navigation satellite system (GNSS), measuring coordinates with different sampling frequencies of on average 0.7 (0.4 Hz).

### 2.3. Measurement protocol

Measurements were performed during the Enschede marathon (42.195 km) in the Netherlands on a typical Dutch spring day with temperatures around 10°C. The course was relatively flat, with about 42 meters of elevation and was performed in relatively open space (i.e., no woods) on the road. The track consisted of two laps of which about 60% of the course was identical between laps. Before the marathon, multiple anthropometric values were measured (body height, hip height, hip width, knee height, ankle height, and shoe length). Sensor-to-segment calibration was performed according to the manufacturer's recommendations (34). Subjects were instructed to run their marathon as planned and not to worry about the equipment.

### 2.4. Data analysis

The data presented in this study are openly available on figshare (https://doi.org/10.6084/m9.figshare.22331686.v1).

#### 2.4.1. Data extraction and computing speed

Sensor data was extracted from the internal memory of the bodypack. Sensor acceleration, angular velocity, and magnetometer data were used to estimate sensor orientations in the software package Xsens MVN Analyze (version 2019.2.1). A scaled biomechanical model was created based on anthropometric measurements, raw sensor data (accelerations and angular velocities), and estimated sensor orientations. Knee flexion angles and pelvis IMU velocity were obtained from this scaled biomechanical model (34). Latitude and longitude coordinates were extracted from the GNSS data. Missing latitude-longitude coordinates were linearly interpolated before speed was computed as the distance between two latitude-longitude coordinates based on the Haversine formula (35). Speeds above 20 km/h were deemed extremely unlikely and replaced with spline interpolation. Speed was then resampled to 240 Hz to match the sampling frequency of the IMUs.

#### 2.4.2. Temporal synchronization

GNSS and IMU data were temporally aligned based on GNSS speed and speed of the pelvis IMU. Pelvis IMU speed was computed as the resultant pelvis IMU velocity obtained from the scaled biomechanical model. GNSS and IMU data were then synchronized by cross-correlating GNSS speed with pelvis IMU speed. Temporal alignment between both systems was visually checked at the start and end of the marathon to ensure that possible differences in internal clocks would not influence temporal alignment. Visual misalignment was present in data of one subject, for which IMU data was resampled based on cross-correlation of the first and last 20% of the data points separately.

#### 2.4.3. Removing walking parts and segmentation

Some participants walked for short periods during the marathon to drink something or due to fatigue. PTA is higher for running than for walking (36). Walking parts were detected and removed based on a minimum of two adjacent outliers in PTA of the right leg. In this case, an outlier was defined as a PTA value of more than four scaled median absolute deviations below the median over the complete marathon. The median absolute deviation is computed as the median of absolute deviations from the median value (37). The median absolute deviation is then scaled by multiplying by 1.48 to approximate the standard deviation as typically reported in literature (38). Additionally, ten strides before and after a walking part were removed to omit the effect of slowing down and increasing speed. After removing the walking parts, data were segmented into time-normalized gait cycles starting with initial contact based on right foot accelerations (39).

#### 2.4.4. Extracting quantities of interest and removing outliers

Quantities of interest were computed for the right legs from all subjects. PTA was defined as the positive acceleration peak in the axial direction of the tibia in a sensor-fixed coordinate system during the first 33% of the gait cycle. Accelerations in the axial direction compared to the resultant acceleration were chosen to better represent the main direction of impact forces in the body. Knee flexion angles were defined as 0° when the leg was fully extended, and flexion resulted in positive values. The knee angle at initial contact was extracted from the first sample of the time-normalized gait cycle. Maximum stance phase knee flexion was defined as the maximum knee angle during the first 33% of the gait cycle. Stride frequency (strides/minute) was based on the time between two right initial contacts. Speed was averaged over the complete gait cycle. The average foot strike angle (i.e., angle between the foot and horizontal in the sagittal plane at initial contact) over the complete marathon was computed to determine the foot strike pattern of subjects (40). Outliers in quantities of interest were defined as values deviating more than four scaled median absolute deviations from the moving median over a window of 500 strides. A relatively large deviation from the median value was chosen to classify outliers to prevent removal of a considerable amount of data and over-smoothing the data. All strides with an outlier in any of the quantities of interest were removed from further analyses.

**Figure 2:**
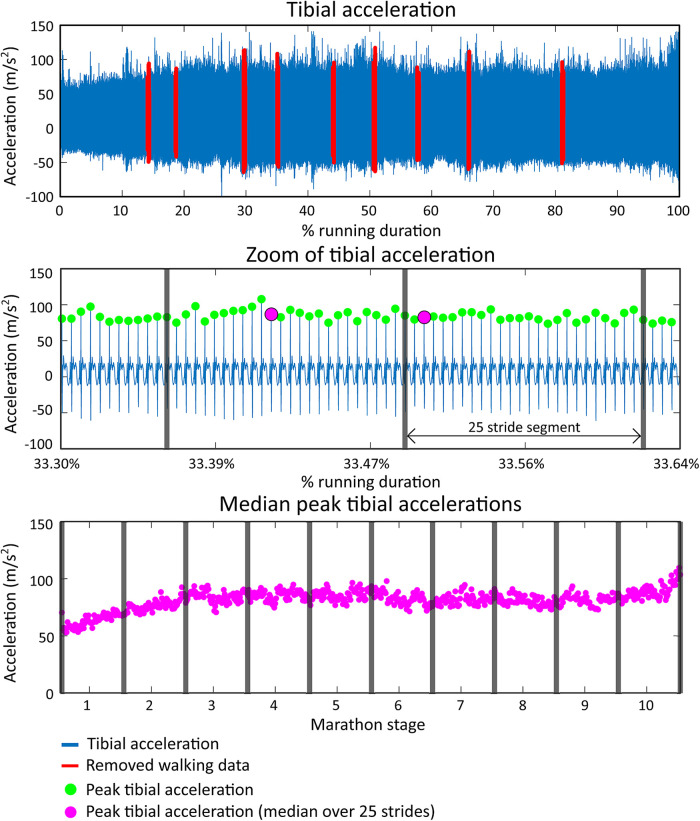
Data extraction shown for peak tibial acceleration (PTA). The top figure shows the tibial acceleration of a representative runner (runner 7) during the full marathon. Walking parts are labeled red and removed from further analysis. The middle figure shows a snapshot of the tibial acceleration signal in which PTAs are shown with green dots. Vertical grey lines show segments of 25 strides from which the median PTA is computed and shown as a pink dot. The bottom figure shows all median PTA values during the marathon. The full marathon duration is divided into ten stages for group-based statistical analyses.

Median values over segments of 25 strides were computed, and outliers were removed (>4 scaled median absolute deviation from moving median over a window of 500 segments) to improve data stability and reduce the amount of data (41). The marathon was divided into ten stages to investigate the effect of marathon stage; each stage was roughly equal to 4 km of running data. Mean values for each stage of the marathon were computed from the earlier defined median values, see Figure 2.

### 2.5. Statistical analysis

Group-based one-way repeated measures ANOVAs were performed to test whether running speed, stride frequency, PTA, knee angles at initial contact, and maximum stance phase knee flexion changed over the different stages of the marathon. The ANOVAs had ten levels (stages of the marathon), and the mean values for each subject for all ten stages were used as input. When a significant effect of marathon stage on one of the quantities of interest was found, *post hoc* tests were used to test which marathon stages differed from each other.

Subject-specific multiple linear regression models were created to test if running speed and stride frequency could predict PTA, knee angles at initial contact, and maximum stance phase knee flexion angles. Median values for all 25 stride segments were used as input for the regression models, and no distinction for marathon stage was made. Intercepts and coefficients from the subject-specific regression equations were used to correct PTA and knee angles for the subject-specific effect of changes in speed and stride frequency. PTA and knee angles were corrected for individual changes in speed by subtracting the individual coefficients for speed (i.e., output of the multiple linear regression model) multiplied with the deviation from the individual mean speed for all segments of 25 strides during the marathon. Corrections for individual changes in stride frequency were performed similarly. Effectively, this method recomputes the quantities of interest as if the speed and stride frequency were equal to the average speed and stride frequency during the whole marathon.

Group-based one-way repeated measures ANOVAs (10-levels) were repeated to test whether speed and stride frequency corrected PTA, knee angles at initial contact, and maximum stance phase knee flexion changed over the different stages of the marathon.

An alpha level of 0.05 was used to determine statistical significance. When applicable, Holm-Bonferroni corrections were applied for all possible 45 *post hoc* pairwise comparisons. Correlations were interpreted as very strong *r* = (0.90, 1.00), strong for *r* = (0.70, 0.89), moderate for *r* = (0.40, 0.69), weak for *r* = (0.20, 0.39) and very weak for *r* = (0.00, 0.19) (42). Statistical analyses were performed in JASP (version 0.16.3).

## 3. Results

Subjects finished the marathon in 3 h and 55 min (30 min), with an average speed of 11.0 (1.5) km/h and an average stride frequency of 85.6 (2.9) strides/minute. Walking parts resulted in the removal of 2.7 (2.1)% of all data points. An average of 19,383 (2,073) gait cycles were measured per runner, of which 8.8 (2.4)% was removed due to outliers. Runners 1 and 5 were classified as non-rearfoot strikers based on a foot contact angle smaller than 8° (40).

### 3.1. Speed and stride frequency

**Figure 3:**
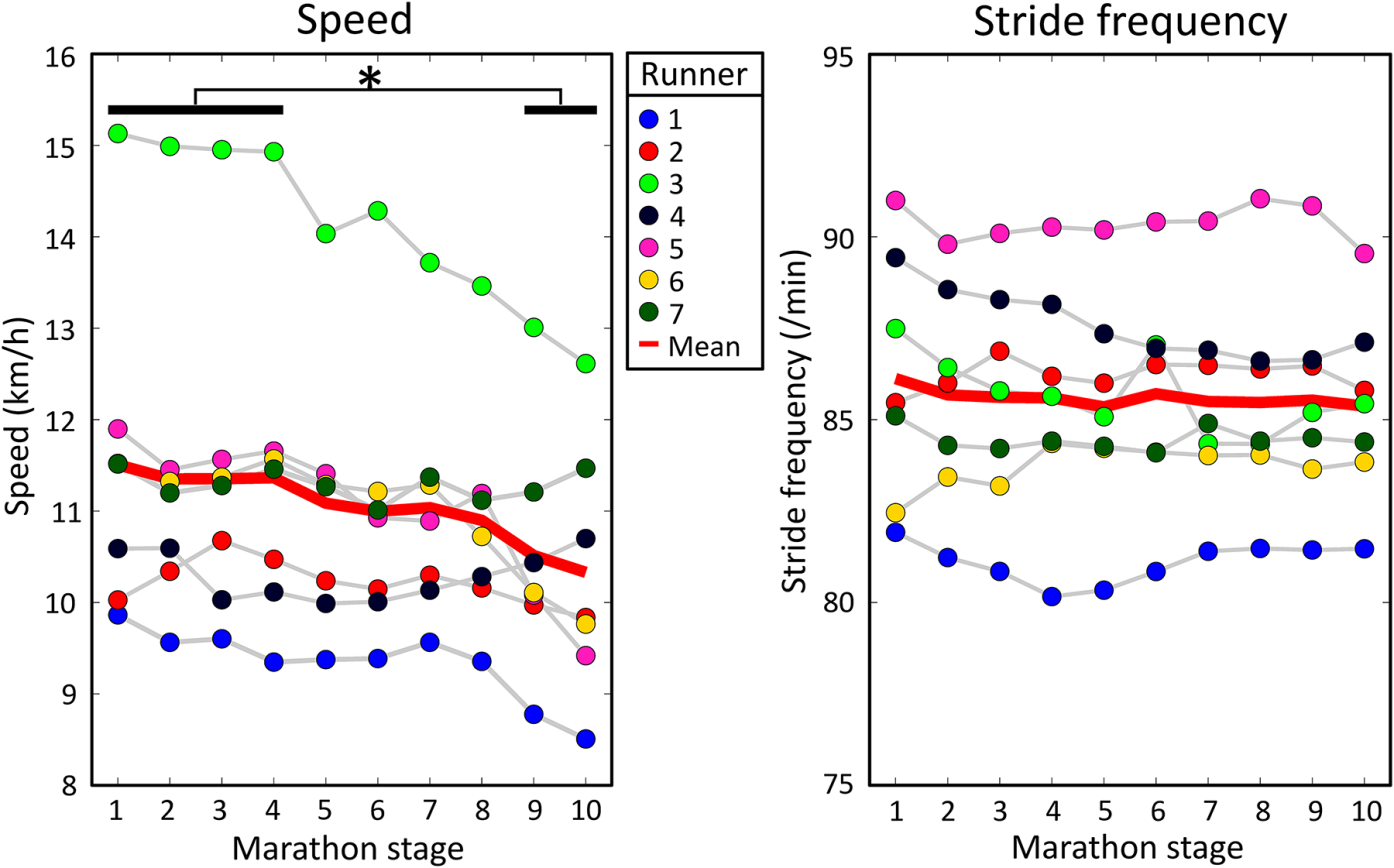
Mean speed and stride frequency for every runner during all marathon stages. The solid red lines show the group means. A significant effect of marathon stage on speed was found. Significant results from *post hoc* analyses are shown by an asterisk at the top of the figure.

There was a statistically significant effect of marathon stage on speed on a group level, F(9,54) = 5.766, *p* < 0.001, see Figure 3. Running speed decreased from 11.5 (1.8) km/h to 10.3 (1.4) km/h between the first and last stages of the marathon. Post-hoc analyses showed that speed during the last two stages was lower than in the first four stages of the marathon. No significant effect of marathon stage on stride frequency was found on a group-level, F(9,54) = 0.725, *p* = 0.684. Speed and stride frequency were weakly correlated on a group level, *r* = 0.21 (0.18).

### 3.2. Peak tibial acceleration

**Figure 4:**
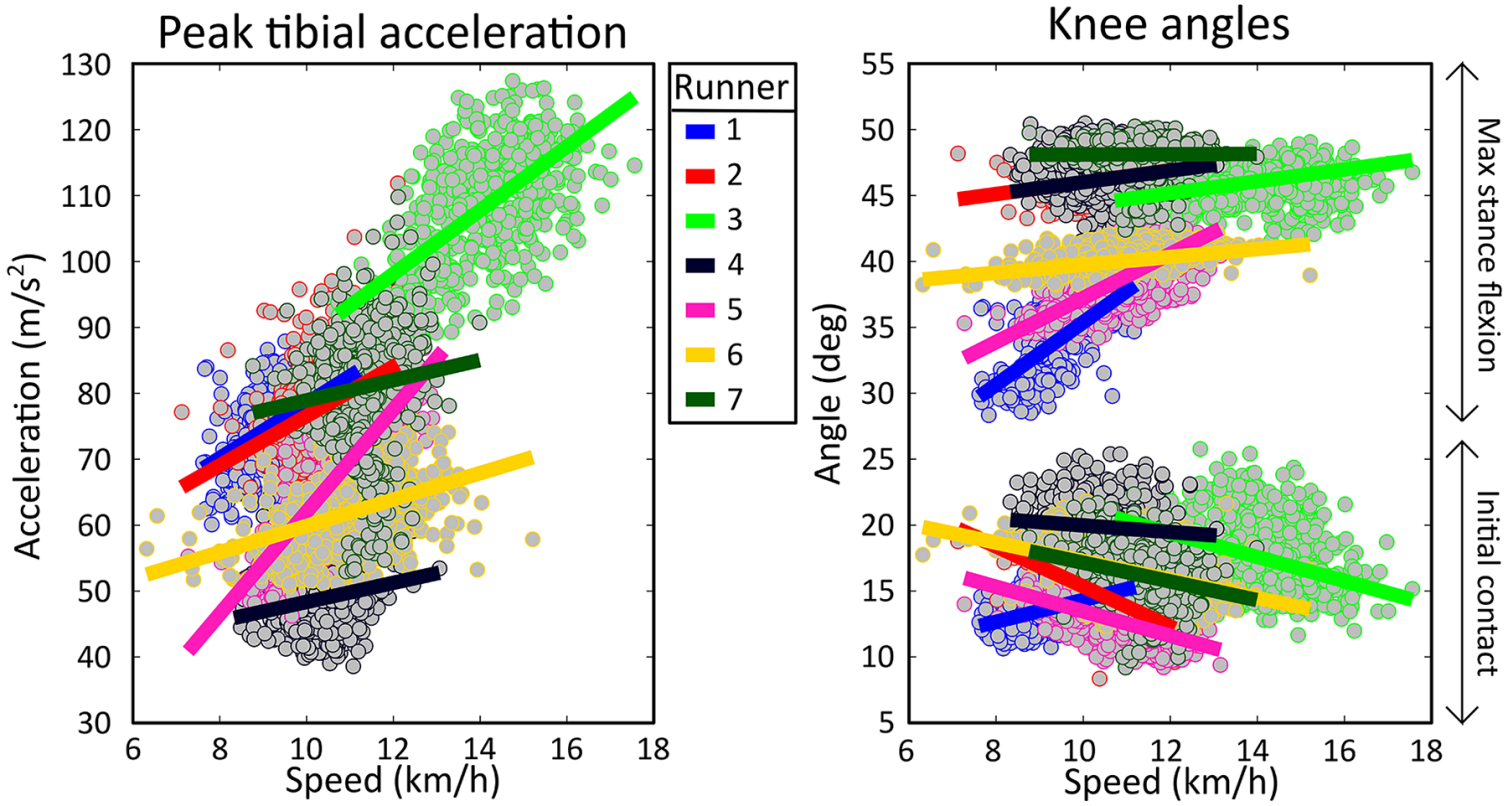
Scatterplot of individual peak tibial acceleration and knee angle values as a function of speed. Each dot represents the median value over a segment of 25 strides during the marathon. Subject-specific linear regressions are shown as solid lines.

**Figure 5:**
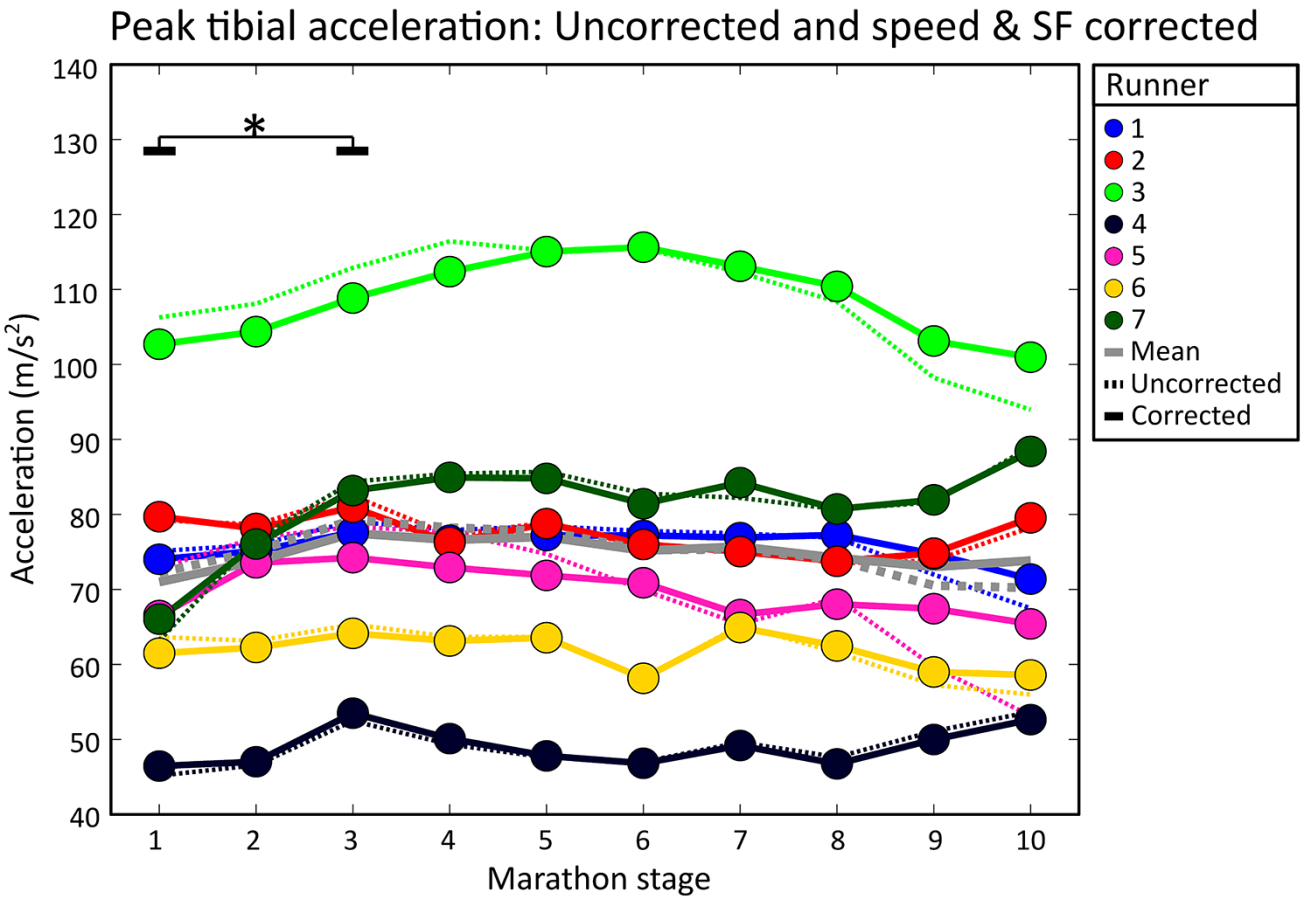
Individual mean peak tibial accelerations during all marathon stages. Dotted lines show uncorrected PTA values (i.e., as measured during the marathon). Solid lines represent speed and stride frequency corrected PTA values. Grey lines show the group means. Significant effects of running duration are shown with an asterisk and black lines. Solid black lines represent significant differences in corrected PTA values.

On a group level, PTA had a moderate positive correlation with speed [*r* = 0.40 (0.24)] and no correlation was present with stride frequency due to large variability between subjects [*r* = −0.09 (0.20)], see Table 1. Subject-specific multiple linear regression equations to predict PTA based on speed and stride frequency were significant for all subjects and explained 26 (18)% of the variance in PTA, see Figure 4. Speed was a significant predictor of PTA for all runners while stride frequency was a significant predictor for all but one runner. On a group level, there was a statistically significant effect of marathon stage on PTA both for uncorrected [F(9,54) = 2.786, *p* = 0.009] and speed and stride frequency corrected values [F(9,54) = 2.316, *p* = 0.028]. However, *post hoc* analyses only showed significant differences in PTA between marathon stages after correcting for speed and stride frequency, see Figure 5. *Post hoc* analysis showed that PTA corrected for speed and stride frequency was higher in the third [77.5 (17.3)] compared to the first stage of the marathon [71.0 (17.5)].

**Table 1:** Left side of table: individual correlations of peak tibial acceleration (PTA) with speed and stride frequency (SF). Right side of table: Individual regression equations to predict PTA based on speed and stride frequency together with the adjusted R-squared value (i.e., explained variance of regression equation). r, Pearson's correlation coefficient; SD, standard deviation; ns, non-significant finding; NRF, non-rearfoot striking subject.

PTA	Correlation		Regression			
	Speed (r)	SF (r)	Intercept	Coefficient speed $\left(\frac{m/s^{2}}{\mathrm{km}/h}\right)$	Coefficient SF $\left(\frac{m/s^{2}}{\mathrm{strides}/\min}\right)$	Adjusted R^2^
Runner 1^NRF^	0.48	−0.22	148.18	4.14	−1.37	0.31
Runner 2	0.33	0.11	65.78	3.75	−0.31^ns^	0.11
Runner 3	0.55	0.11	106.14	4.83	−0.77	0.32
Runner 4	0.17	−0.21	115.32	1.45	−0.93	0.08
Runner 5^NRF^	0.79	0.12	23.27^ns^	7.76	−0.43	0.62
Runner 6	0.44	−0.17	114.41	2.00	−0.89	0.21
Runner 7	0.06^ns^	−0.39	479.17	1.53	−4.92	0.16
Mean (SD)	0.40 (0.24)	−0.09 (0.20)	150.32 (150.50)	3.64 (2.26)	−1.37 (1.60)	0.26 (0.18)

### 3.3. Knee angle at initial contact

**Figure 6:**
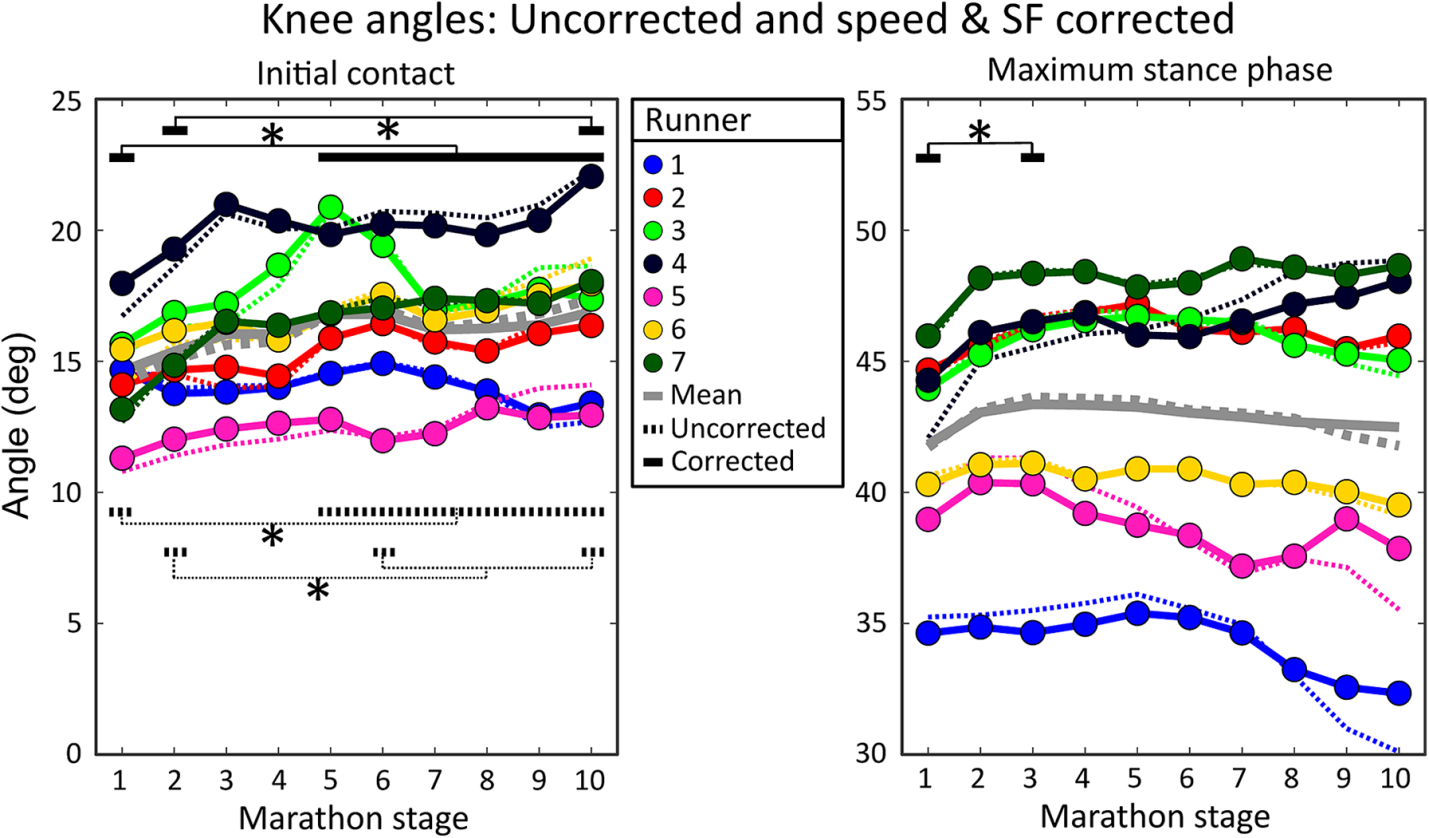
Individual mean knee angles during all marathon stages. The left figure shows knee angles at initial contact, while the right figure shows maximum stance phase knee flexion angles. Dotted lines show uncorrected knee angles (i.e., as measured during the marathon). Solid lines represent speed and stride frequency corrected knee angles. Dotted black lines represent significant differences in uncorrected knee angles while solid black lines represent significant differences in knee angles corrected for the effect of changes in speed and stride frequency. Grey lines show the group means. Significant effects of marathon stage are shown with an asterisk and black lines.

On a group level, knee angles at initial contact showed a weak negative correlation with speed [*r* = −0.24 (0.30)] and no correlation was present with stride frequency due to large variability between runners [*r* = −0.03 (0.28)], see Table 2. Subject-specific multiple linear regression equations to predict knee angles at initial contact based on speed and stride frequency were significant for all subjects and explained 20 (10)% of the variance in knee angles at initial contact, see Figure 6. Speed was a significant predictor of knee angles at initial contact for all runners while stride frequency was a significant predictor for all but two runners. On a group level, there was a statistically significant effect of marathon stage on both corrected [F(9,54) = 5.136, *p* < 0.001] and uncorrected knee angles at initial contact [F(9,54) = 7.227, *p* < 0.001]. *Post hoc* analyses showed that knee angles at initial contact during later stages of the marathon were significantly higher than during the first stages of the marathon, see Figure 6.

**Table 2:** Left side of table: individual correlations of knee angles at initial contact (IC) with speed and stride frequency (SF). Right side of table: Individual regression equations to predict knee angles at IC based on speed and stride frequency together with the adjusted R-squared value (i.e., explained variance of regression equation). r, Pearson’s correlation coefficient; SD, standard deviation; ns, non-significant finding; NRF, non-rearfoot striking subject.

Knee IC	Correlation		Regression			
	Speed (r)	SF (r)	Intercept	Coefficient speed $\left(\frac{\deg}{\mathrm{km}/h}\right)$	Coefficient SF $\left(\frac{\deg}{\mathrm{strides}/\min}\right)$	Adjusted R^2^
Runner 1^NRF^	0.37	0.02 ^ns^	10.08	0.83	−0.05 ^ns^	0.13
Runner 2	−0.44	−0.24	44.34	−1.52	−0.16 ^ns^	0.19
Runner 3	−0.29	0.06 ^ns^	2.04 ^ns^	−0.91	0.33	0.12
Runner 4	−0.15	−0.42	78.54	−0.25	−0.64	0.18
Runner 5^NRF^	−0.51	0.19	−17.90	−0.93	0.45	0.36
Runner 6	−0.43	0.40	−45.22	−0.70	0.83	0.32
Runner 7	−0.25	−0.21	67.32	−0.71	−0.51	0.09
Mean (SD)	−0.24 (0.30)	−0.03 (0.28)	19.89 (45.40)	−0.60 (0.73)	0.04 (0.53)	0.20 (0.10)

### 3.4. Maximum stance phase knee angles

On a group level, maximum stance phase knee angles had a weak positive correlation with speed [*r* = 0.32 (0.27)] and a weak negative correlation with stride frequency [*r* = −0.21 (0.28)], see Table 3. Subject-specific multiple linear regression equations to predict maximum stance phase knee angles based on speed and stride frequency were significant for all subjects and explained 30 (20)% of the variance in maximum stance phase knee angles, see Figure 6. Speed was a significant predictor for maximum stance phase knee angles for all runners while stride frequency was a significant predictor for all but one runner. On a group level, marathon stage had no statistically significant effect on maximum stance phase knee flexion [F(9,54) = 1.770, *p* = 0.096]. After correcting knee angles for subject-specific effects of speed and stride frequency, a significant effect of marathon stage on maximum stance phase knee flexion was found [F(9,54) = 2.294, *p* = 0.029]. *Post hoc* analyses showed that the maximum stance knee flexion corrected for speed and stride frequency was significantly higher in the third [43.4 (4.9)] compared to the first stage of the marathon [41.8 (4.0)], see Figure 6.

**Table 3:** Left side of table: individual correlations of maximum stance phase knee angles with speed and stride frequency (SF). Right side of table: Individual regression equations to predict maximum stance phase knee angles based on speed and stride frequency together with the adjusted Rsquared value (i.e., explained variance of regression equation). r, Pearson’s correlation coefficient; SD, standard deviation; ns, non-significant finding; NRF, non-rearfoot striking subject.

Knee stance	Correlation		Regression			
	Speed (r)	SF (r)	Intercept	Coefficient speed $\left(\frac{\deg}{\mathrm{km}/h}\right)$	Coefficient SF $\left(\frac{\deg}{\mathrm{strides}/\min}\right)$	Adjusted R^2^
Runner 1^NRF^	0.59	−0.27	76.58	2.37	−0.80	0.48
Runner 2	0.31	0.25	25.13	0.45	0.19	0.11
Runner 3	0.22	−0.22	62.92	0.45	−0.27	0.17
Runner 4	0.03^ns^	−0.69	152.86	0.44	−1.27	0.49
Runner 5^NRF^	0.69	−0.08	63.90	1.67	−0.48	0.54
Runner 6	0.42	−0.17	48.46	0.30	−0.14	0.20
Runner 7	−0.04^ns^	−0.30	89.40	0.01^ns^	−0.49	0.09
Mean (SD)	0.32 (0.27)	−0.21 (0.28)	74.18 (40.27)	0.81 (0.86)	−0.47 (0.47)	0.30 (0.20)

## 4. Discussion

This research aimed to quantify and correct for the subject-specific effect of changes in running speed and stride frequency on impact-related running mechanics during a fatiguing outdoor run. In line with our first hypothesis, speed decreased throughout the marathon, however, no effect of marathon stage on stride frequency was found. Running speed and stride frequency explained, on average 20%–30% of the variance in PTA, knee angles at initial contact, and maximum stance phase knee flexion while running in an uncontrolled setting. Regression coefficients for speed and stride frequency varied strongly between subjects, supporting our second hypothesis. Our third hypothesis was not supported for PTA and knee angles at initial contact, but was supported by a significant change in maximum stance phase knee flexion corrected for changes in speed and stride frequency, while uncorrected values showed no significant change during the marathon.

### 4.1. Speed and stride frequency

Running speed significantly decreased during the marathon. A decrease in speed during a marathon is typically found (19, 20, 43, 44) and is likely caused by fatigue, although race strategy can also play a role. Stride frequency did not show a significant effect of marathon stage and was weakly correlated with speed, indicating that, similar to previous studies, the speed reduction is generally caused by a decrease in stride length instead of stride frequency (19, 45). The significance of predictors, regression equations, and explained variances differed between subjects. Differences might be caused by differences in body weight, ankle angle at initial contact (46), foot strike pattern (20), individual differences in adaptations to speed by increasing step length vs. stride frequency, differences in the tolerance to effects of fatigue and differences in the capacity to sustain a stable gait pattern over a range of speeds. Even though stride frequency did not change on a group level, adding stride frequency to the regression models resulted in significantly better predictions for almost all runners, emphasizing the benefits of subject-specific analysis vs. group-based analysis.

### 4.2. Peak tibial acceleration

Average group-based PTA values (81.2 ± 8.4 m/s^2^) showed a significant main effect of marathon stage, although *post hoc* analyses showed no differences between marathon stages for uncorrected values. PTA values were within the range found in literature (49.1–116.7 m/s^2^) (8, 20, 47). The correlations between PTA and speed [*r* = 0.40 (0.24)] during a marathon were similar to the correlations between resultant PTA and speed in controlled settings (*r* = 0.42) (24). Subject-specific multiple linear regressions showed that, on average, PTA increased with 3.6 m/s^2^ for every 1 km/h increase in speed, although subject-specific coefficients ranged from 1.5 to 7.8 m/s^2^. The speed coefficient of PTA was between 4.1 and 6.7 m/s^2^ in controlled settings (23, 48). The speed coefficient to predict PTA in our study was generally lower than in laboratory-based studies, possibly due to the inclusion of stride frequency or external influences like fatigue. Foot strike pattern has been shown to influence the speed coefficient of PTA during a marathon. Rearfoot striking runners showed higher speed coefficients (12.8 m/s^2^) than midfoot striking runners (7.0 m/s^2^), while no significant speed coefficient was found for forefoot striking runners (20). In our study, the two non-rearfoot striking runners (subjects 1 and 5) had amongst the highest speed coefficients, which is possibly an effect of group- vs. subject-based analysis. The regression equation explained, on average, 26 (18)% of the variance in PTA. Although relatively low, it is higher than the 19% of explained variance in resultant PTA found in laboratory-based studies (24). To accurately predict PTA in outdoor environments, more variables are needed in the multiple linear regression equation (e.g., knee angle at initial contact), but for the scope of this paper, we were solely interested in the explained variance by speed and stride frequency. After correcting PTA for the subject-specific effects of speed and stride frequency, *post hoc* tests showed a significant increase in PTA between the first and third stages of the marathon. While this increase in PTA occurred very early on in the marathon, it is not uncommon for peak accelerations to increase during early stages of a fatiguing protocol (49–51). An increase in PTA corrected for changes in speed and stride frequency could indicate a decrease in the runner's capacity to attenuate shocks. Alternatively, the effective mass (i.e., the portion of body mass that is decelerated upon ground contact) can decrease with an increased rearfoot or knee flexion angle and increase with a more vertical lower leg at initial contact (46, 52). A smaller effective mass is easier to accelerate, which results in higher leg accelerations when similar ground reaction forces are applied. High PTA values have long been thought to be an indicator of injury risk based on retrospective studies showing that PTA was higher in previously injured compared to uninjured runners (53, 54). The repetitiveness of high forces on tissues inside the body are thought to be related to the development of running injuries (55). However, PTA is uncapable to represent tibial bone loading since it does not represent forces caused by muscle contractions in the body (9, 10). Hence, high PTA values are no indicator of injury risk on their own but do provide information about a combined effect of impact forces and running technique on the body during running.

### 4.3. Knee angles

Average knee angles at initial contact (16.1 (2.5)°) and maximum stance phase knee angles (42.9 (5.1)°) were within the range reported in literature, respectively 9.5°–19.5° and 31.0°–56.2° (4, 26, 52, 56, 57). Knee angles at initial contact showed a negative weak and very weak correlation with speed and stride frequency, indicating more knee extension with higher speeds and stride frequencies. Previously, the knee flexion angle at initial contact remained similar (26) or increased with speed (58), although the range of speeds included was drastically higher than those found during the marathon. A decrease in knee angle at initial contact with an increase in speed might be a strategy to increase stride length by increasing leg extension. Knee angles at initial contact corrected for subject-specific effects of changes in speed and stride frequency showed a similar increasing pattern during the marathon compared to uncorrected values. Knee angles at initial contact have been found to increase with fatigue in controlled settings (12, 52, 57), possibly to decrease vertical ground reaction forces (46) at a higher metabolic cost (59). Hence, the increase in knee angles at initial contact during a marathon is not solely an effect of changes in speed and stride frequency but is likely a result of fatigue.

Maximum stance phase knee angles had a weak positive correlation with speed and a weak negative correlation with stride frequency, indicating that the stance phase shortens at higher stride frequencies, resulting in less knee flexion during stance (25). An increase in knee flexion with an increase in speed has been shown previously (26) and might be caused by higher forces on the body that need to be absorbed at higher speeds. The average increase in maximum stance phase knee flexion of 0.8° for every 1 km/h increase in speed is similar to previous findings in controlled settings (0.7°) (26). Maximum stance phase knee flexion angles corrected for changes in speed and stride frequency reveal a significant increase between the first and third stages of the marathon that is not present in uncorrected values. Although a statistically significant difference was found after correcting maximum stance phase knee angles for subject-specific effects of speed and stride frequency, the sample size was small with large variability and the clinical relevance of this finding might be limited. Further research should investigate the effect of speed and stride frequency on knee angles in more runners to investigate the clinical relevance and repeatability of this finding. However, an increase in maximal stance knee flexion could indicate an increase in stride length (60), knee extensor strength loss, or a reduced tolerance to imposed stretch loads with fatigue (44, 61). Despite relatively small explained variances of regression equations for knee angles, subject-specific corrections for changes in speed and stride frequency on knee angles significantly influenced the interpretation of mechanical changes during a marathon.

### 4.4. Fatigue

Subjects likely experienced high levels of fatigue toward the end of the marathon. Running-induced fatigue typically increases PTA (12), knee flexion at initial contact (12) and tends to increase maximal stance phase knee flexion (52, 57, 62). Both speed and fatigue have been positively associated with PTA and maximum stance phase knee angles (8, 12, 26, 52). Fatigue might have caused lower speed coefficients for PTA and maximum stance phase knee angles than expected without the influence of fatigue. Since subjects generally ran slower at the end of the marathon, PTA and maximum knee angles possibly decreased less with a decrease in speed towards the end of the marathon due to fatigue. Therefore, the influence of speed and stride frequency on running mechanics in an uncontrolled environment might be larger than shown in this study. To omit the effect of fatigue, we could have taken data from the start of the marathon, defined linear regression equations from data in an unfatigued state, and applied a correction to the remainder of the data, similar to Clermont et al. (63). However, most runners will experience some level of fatigue during their runs, making relationships solely based on unfatigued data invalid. Hence, we deliberately included data from an unfatigued to a fully fatigued state to create subject-specific relationships with better ecological validity.

### 4.5. Limitations

Collecting data in an uncontrolled environment is both a benefit and a shortcoming of this study. The benefit is that runners were measured in the actual environment where they typically run without any constrictions that a laboratory setting or a treadmill would impose on their gait pattern. However, we investigated the effects of speed and stride frequency on multiple mechanical quantities. At the same time, many other external influences could have played a role, such as running surface, fatigue, other runners, or distractions. The explained variance of quantities of interest can be improved by incorporating additional variables into the regression equation. However, for the scope of this paper, we were only interested in how much of the variance in included quantities could be explained by changes in speed and stride frequency.

The limited number of subjects in this study might have led to an underpowered group-based analyses to compare the effect of marathon stage on both corrected and uncorrected PTA and knee angle values. However, the large variability between runners, as demonstrated in the running speed, and speed coefficients for PTA and knee angles, emphasized the need for subject-specific analysis and corrections of the gait pattern, as demonstrated in this research.

Measurement equipment could have influenced the findings of this study in multiple ways. Different sports watches were used which could result in varying sampling frequencies and accuracies of the measured running speed. A lower sampling frequency could lead to allocation of varying PTA or knee angle values to a single running speed or stride frequency, therefore influencing the speed and stride frequency coefficients. Additionally, a low sampling frequency or GNSS signal loss could lead to high measured running velocities (>20 km/h). These high velocities were deemed unlikely and replaced by spline interpolation, potentially underestimating the actual running velocity. However, by computing quantities over segments of 25 strides, the running speed during each segment was computed based on multiple measurements, decreasing the influence of a low running speed sampling frequency and varying accuracies of sports watches on the outcomes of this study. For future studies we would recommend to compute running speed based on IMU signals.

Furthermore, PTA was measured with an IMU fixed to the skin. Skin-mounted IMUs are prone to soft-tissue artefacts as they can move with respect to the underlying bone, resulting in measurement errors (8). IMU displacement with respect to the body could have occurred due to sweating which would have influenced the sensor-to-segment calibration and output of the IMUs. However, IMUs seemed to be properly attached after the marathon and no sudden changes of a sensor becoming loose were found in the data.

### 4.6. Practical implications

This study showed that running speed and stride frequency have a subject-specific relationship with PTA, knee angles at initial contact, and maximum stance phase knee flexion. Correcting for these relationships influences the interpretation of changes in mechanical quantities while running in an uncontrolled environment and allows for comparisons of mechanical quantities between runs at different running speeds. Many wearable devices provide feedback on peak accelerations to improve the gait pattern (29–31). Typically, feedback is provided on PTA values above a certain generic threshold. PTA values above this threshold which are caused by an increase in running speed or a decrease in stride frequency provide information about the absolute PTA value. The absolute PTA value can easily be lowered by changing running speed or stride frequency. In addition, speed and stride-frequency corrected PTA values provide information about relative PTA values. Speed and stride frequency corrected PTA values that are higher than expected at the current speed and stride frequency can warn runners for suboptimal changes in their gait pattern. Hence, changes in speed and stride frequency corrected PTA values provide information about a shift in the human body while performing a similar task (i.e., running at the same speed with the same stride frequency). A subject-specific model of the relationship between speed, stride frequency and quantities of interest based on multiple runs in different conditions would allow for comparisons of mechanical quantities between runs independent of running speed and stride frequency to better monitor progression of a runner. While this study demonstrated the effect of subject-specific corrections on PTA and knee angles, similar corrections can be made for other quantities of interest. For instance for biomechanical risk factors for overuse injuries like knee stiffness, peak hip adduction, ankle eversion or pelvic drop angles (7, 64, 65). Hence, we advise investigating and correcting for subject-specific regression equations for all quantities of interest when measuring, comparing and providing feedback on running mechanics in an uncontrolled environment.

## Conclusions

In this study, we quantified and corrected for the subject-specific effect of changes in running speed and stride frequency on impact-related running mechanics during a fatiguing outdoor run. Subject-specific corrections through multiple linear regression equations revealed a significant effect of marathon stage on maximal stance phase knee flexion, which was previously masked by changes in speed and stride frequency. The effect of marathon stage on PTA and knee angles at initial contact changed after correcting for changes in speed and stride frequency. Hence, speed and stride frequency influence the interpretation of changes in mechanical quantities in a subject-specific manner when running in an uncontrolled environment. Subject-specific effects of speed and stride frequency on quantities of interest should be investigated and corrected when interpreting, or providing feedback on, running mechanics in an uncontrolled environment.

## Data Availability

The accompanying dataset for this study is publicly available. This data can be found here: https://doi.org/10.6084/m9.figshare.22331686.v1.
